# Support Vector Machine Models for Cancer Detection and Related Clinical Applications Using Omics and Omics‐Adjacent Data: A Systematic Review

**DOI:** 10.1155/bmri/5935532

**Published:** 2026-09-29

**Authors:** Zhina Mohamadi, Rozhina Mohammadi, Erfan Abtahi, Zahra Sadat Shayegh, Mehrafarin Ataei Kachouei, Amin Fakhar, Mohammad Mahdi Shirani, Mohammadhosein Malekian, Amir Zinatshoar, Mahdi Biglari, Fatemeh Rezaei, Armin Zarinkhat

**Affiliations:** ^1^ Faculty of Medicine, Kermanshah University of Medical Sciences, Kermanshah, Iran, kums.ac.ir; ^2^ Faculty of Electrical Engineering, Amirkabir University of Technology, Tehran, Iran, aut.ac.ir; ^3^ Medical Branch, Islamic Azad University, Tehran, Iran, tiau.ac.ir; ^4^ Tonekabon Azad University of Medical Sciences, Mazandaran, Iran; ^5^ Faculty of Medicine, Isfahan University of Medical Sciences, Isfahan, Iran, mui.ac.ir; ^6^ Department of Orthopedics, Shafa Hospital, Iran University of Medical Sciences, Tehran, Iran, iums.ac.ir; ^7^ Kerman Medical Branch, Islamic Azad University, Kerman, Iran, tiau.ac.ir

**Keywords:** cancer, machine learning, molecular omics, omics-adjacent data, support vector machine, systematic review

## Abstract

**Background:**

Support vector machines (SVMs) have been widely applied to high‐dimensional molecular and related biomedical data for cancer detection and other clinical classification tasks. The evidence spans diverse cancer types, specimens, platforms, objectives, and validation designs, limiting direct comparison.

**Methods:**

We systematically reviewed studies evaluating SVM‐based models using molecular omics or high‐dimensional omics‐adjacent data for cancer‐related clinical applications. Article‐level characteristics, SVM implementation, feature selection, validation, comparator models, and reported performance measures were extracted. Risk of bias was evaluated with the Prediction Model Risk Of Bias Assessment Tool (PROBAST). Because the studies did not estimate a common clinically interpretable quantity, no meta‐analysis, pooled estimate, median performance comparison, or statistical subgroup comparison was performed. Findings were synthesized descriptively by data modality, clinical task, validation approach, comparative modeling, and methodological quality.

**Results:**

Seventy‐five studies met the revised eligibility criteria. Sixty used molecular omics data; the other 15 used omics‐adjacent inputs (10 spectroscopy‐derived, two sensor derived, two mixed‐data, and one imaging‐derived). Cancer detection was the leading application. Internal validation alone was reported in 60 studies; eight combined internal and external validation, four relied on external validation only, two reported no validation, and one remained unclear. Seventy‐two studies contributed 544 article‐level SVM performance entries. In a separate PROBAST assessment, 72 studies were rated at high overall risk of bias and three at unclear risk.

**Conclusions:**

SVM‐based models have been studied across a broad range of cancer‐related applications, but the evidence is heterogeneous, predominantly internally validated, and frequently at high risk of bias. A single summary performance estimate would be misleading. This review maps the evidence and identifies cancer‐specific, modality‐specific, and externally validated questions suitable for future focused studies.

## 1. Introduction

Cancer remains a major cause of morbidity and mortality worldwide, with more than 19 million new cases and almost 10 million deaths estimated for 2020 [1]. Although diagnostic and screening approaches continue to improve, clinically useful detection requires adequate discrimination, reproducibility, and validation in relevant populations [2]. Molecular omics platforms, including genomics, transcriptomics, proteomics, metabolomics, and epigenomics, can characterize cancer‐associated biological variation at high dimensionality [3–5]. Such datasets often contain many more candidate features than observations and therefore require careful preprocessing, feature selection, and model validation [6]. SVMs are margin‐based supervised learning methods that can accommodate high‐dimensional data and nonlinear decision boundaries through kernel functions [7]. They remain common in cancer‐omics studies because they can be effective in small‐sample, high‐feature settings, although their apparent performance is strongly influenced by feature‐selection timing, hyperparameter tuning, class balance, resampling design, and the independence of the test data [8, 9]. In this review, molecular omics comprised genomics, transcriptomics, proteomics, metabolomics, epigenomics, glycomics, lipidomics, immunomics, and the integration of two or more distinct molecular omics layers within a single analytical model. High‐dimensional spectroscopy‐derived, sensor‐derived, imaging‐derived, and mixed analytical inputs were classified separately as omics‐adjacent. Radiomics was not treated as a molecular omics layer. The term multiomics was reserved for studies integrating at least two distinct molecular omics layers in model development, rather than studies using one modality or combining molecular data with imaging or routine clinical variables. The review is aimed at characterizing SVM‐based cancer models across these data families, including clinical application, validation strategy, feature‐selection and modeling procedures, reported performance, comparison with alternative algorithms, and methodological quality. The focus on SVM was prespecified because the method has been repeatedly used as a primary classifier and benchmark in high‐dimensional cancer research; the review does not assume that SVM is superior to newer machine‐learning or deep‐learning methods.

## 2. Methods

### 2.1. Protocol and Reporting

The review followed PRISMA 2020 [10]. A completed PRISMA 2020 checklist is supplied as File S2. The protocol was registered on the Open Science Framework [11] (10.17605/OSF.IO/7VN5X), and an earlier version of the manuscript was posted as a preprint [12]. The registered protocol and preprint retain the original title and broader terminology. After peer review and a complete article‐level audit, we revised the title and operational scope. Specifically, we removed the term implementation, restricted multiomics to models that integrated at least two distinct molecular omics layers, and classified eligible high‐dimensional nonmolecular inputs as omics‐adjacent. These postregistration amendments are reported here and in File S1; the historical titles of the OSF record and preprint were not altered.

### 2.2. Information Sources and Search Strategy

PubMed/MEDLINE, Scopus, Web of Science, and Google Scholar were searched from database inception to August 4, 2024. Search concepts covered SVM, cancer or malignancy, and omics or high‐dimensional biomarker data. Google Scholar was used as a supplementary source with the prespecified allintitle query; all 14 returned records were screened, so no arbitrary first‐*N* cutoff was applied. Full database‐specific Boolean strategies are provided in Table S1.

### 2.3. Screening and Eligibility

Screening was conducted in Rayyan [13]. A seven‐reviewer screening team (Zhina Mohamadi, Erfan Abtahi, Zahra Sadat Shayegh, Amin Fakhar, Amir Zinatshoar, Mahdi Biglari, and Fatemeh Rezaei) assessed titles and abstracts and subsequently evaluated potentially eligible full texts. Every record was independently assessed by at least two reviewers. Disagreements were first discussed to reach consensus. When consensus could not be reached, Armin Zarinkhat independently reviewed the disputed record and made the final eligibility decision in his role as the subject‐matter expert. A formal chance‐corrected agreement statistic, such as Cohen′s kappa, was neither prespecified nor calculated; no retrospective estimate is reported. Eligible reports were English‐language original studies of human malignancy that evaluated an SVM as a final classifier or as a defined component of an eligible model. For eligibility classification, molecular omics data comprised genomic, transcriptomic, epigenomic, proteomic, metabolomic, glycomic, lipidomic, immunomic, and integrated multiomics measurements. Prespecified omics‐adjacent inputs included spectroscopy‐derived molecular fingerprints, sensor outputs, mixed molecular‐clinical features, and the single imaging‐derived model retained in the audited evidence base. Conventional radiomics based solely on imaging features was not classified as molecular omics and was excluded unless it met the prespecified omics‐adjacent criteria. Multiomics was reserved for models integrating at least two distinct molecular omics layers. Studies were also excluded when SVM was used solely for feature selection, solely as a comparator without extractable SVM‐specific results, or when inputs were limited to low‐dimensional routine clinical or laboratory variables. The English‐language restriction was prespecified but introduces language bias.

### 2.4. Data Extraction and Outcome Organization

Data extraction was conducted by a seven‐reviewer team (Mehrafrin Ataei Kachouei, Moham mad Mahdi Shirani, Mohammadhosein Malekian, Amir Zinatshoar, Mahdi Biglari, Rozhina Mo hammadi, and Fatemeh Rezaei) using a structured article‐level extraction form. The extracted entries were reconciled article by article during the final revision audit and retained in File S1. The audit recorded study design, population, cancer type, clinical task, data family, molecular layer, specimen, platform, features, SVM role and kernel, hyperparameters, feature selection and its timing, comparator models, validation design, calibration, clinical utility analysis, reported performance, risk of leakage or overfitting, demographic or fairness reporting, deployment status, and data or code availability. Two geographic variables were retained separately. First_Author_Affiliation_Country denotes the country of the first author′s first listed institutional affiliation and serves as a bibliometric descriptor. Study_Data_Geography captures the reported origin of participants, biospecimens, development cohorts, validation cohorts, or source datasets and is the variable relevant to transportability. Clinical tasks were retained as reported and organized into cancer detection, early detection or screening, differential diagnosis, stage or subtype classification, diagnostic and prognostic applications, treatment‐related prediction, and combined tasks. Internal validation included crossvalidation, leave‐one‐out procedures, bootstrapping, repeated random splitting, or held‐out subsets from the same source population. External validation required evaluation in an independent cohort, institution, dataset, or source population not used for model development or feature selection [14–17]. Insufficiently described designs were classified as unclear.

### 2.5. Risk of Bias

Four reviewers (Erfan Abtahi, Mehrafrin Ataei Kachouei, Mohammad Mahdi Shirani, and Zahra Sadat Shayegh) independently appraised each included study with the Prediction Model Risk Of Bias Assessment Tool (PROBAST) across participants, predictors, outcome, and analysis domains [18]. During revision, study‐level judgments were harmonized against the article‐level evidence. Risk‐of‐bias judgments were integrated into the narrative interpretation rather than used to generate pooled estimates.

### 2.6. Synthesis Approach

The studies differed substantially in cancer type, clinical task, case and control definitions, specimen, analytical platform, feature‐selection procedure, SVM configuration, threshold selection, validation design, and reported outcome measure. Diagnostic, prognostic, screening, staging, subtype‐classification, and treatment‐related models did not estimate a common quantity. Consequently, we did not calculate a pooled AUC, sensitivity, specificity, mean or median performance, or formal between‐group comparison. Findings were retained at article level and synthesized descriptively; counts describe the evidence base rather than comparative effectiveness. Comparator‐related information was assessed for every included study. A universal best/comparable/inferior tally was not imposed because comparator algorithms, optimization procedures, validation sets, outcomes, and metrics varied, and many articles did not report a directly interpretable paired comparison. Combining those contrasts would have produced a deceptively simple summary of nonequivalent evidence.

### 2.7. Use of Artificial Intelligence Tools

During manuscript revision, the authors performed language editing, editorial restructuring, crossdocument consistency checking, LaTeX formatting, preparation of submission materials, and troubleshooting of reproducible figure‐generation code. Where needed, OpenAI ChatGPT 5 and Anthropic Claude were used as assistive tools for these editorial, formatting, consistency checking, and code‐support tasks. These tools were not used for study selection, eligibility decisions, data extraction, data synthesis, statistical analysis, PROBAST judgments, or scientific interpretation. Figures were generated programmatically from the final author‐verified dataset using R and TikZ; no generative image model was used to create or alter figure content. All AI‐assisted suggestions and code changes were critically reviewed, tested, verified, and approved by the authors, who take full responsibility for the final manuscript, code, and figures.

## 3. Results

### 3.1. Study Selection

The searches identified 3482 records: 677 from PubMed/MEDLINE, 642 from Web of Science, 2149 from Scopus, and 14 from Google Scholar. Before screening, 1142 duplicate records and 628 non‐English or nonjournal article records were removed, leaving 1712 records for title and abstract screening. Of these, 1609 were excluded and 103 reports were sought for retrieval. Seven reports could not be retrieved because the full text was unavailable, leaving 96 reports for eligibility assessment. After application of the revised eligibility criteria, 21 reports were excluded and 75 studies were included in the systematic review (Figure 1).

**Figure 1:**
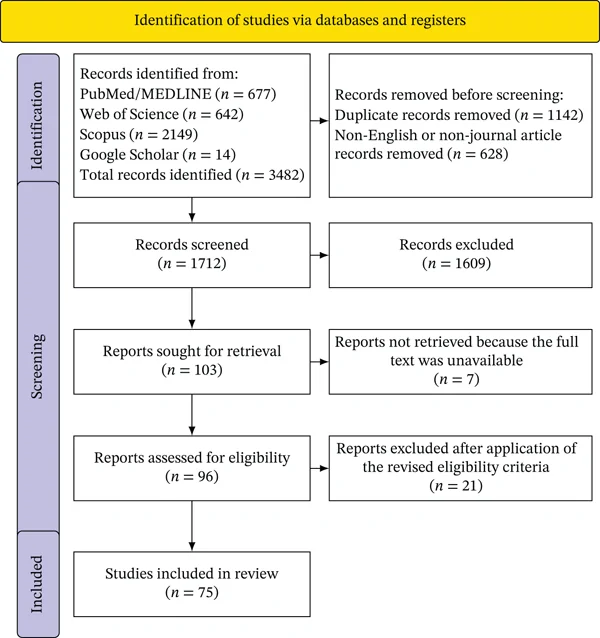
PRISMA‐style study‐selection flow. The searches identified 3482 records. After removal of 1142 duplicate records and 628 non‐English or nonjournal article records, 1712 records were screened. Following title‐and‐abstract screening, 103 reports were sought for retrieval; seven full texts were unavailable, leaving 96 reports for eligibility assessment. Twenty‐one reports were excluded after application of the revised eligibility criteria, and 75 studies were included in the systematic review.

### 3.2. Study Characteristics and Clinical Applications

Of the 75 included studies, 60 used molecular omics data. Fifteen used omics‐adjacent inputs: 10 spectroscopy‐derived, two sensor‐derived, two mixed‐data, and one imaging‐derived study. Under the strict definition used in the revision, only two studies represented true multiomics integration; other studies used one molecular layer or analyzed multiple layers separately. Cancer detection was the most common reported task (*n* = 30), followed by multiple tasks (*n* = 10). The multiple tasks category was reserved for articles that examined several clinical objectives without fitting a single prespecified combined category. Early detection or screening, differential diagnosis, and combined diagnostic‐prognostic objectives each accounted for eight studies; two studies addressed stage classification and one addressed subtype classification. Eight further studies each represented a distinct combined or specialized task. Their complete article level classifications are provided in Table S2 and File S1 (Figure 2). The article‐level selection counts and summary characteristics of the included evidence base are summarized in Table 1.

**Figure 2:**
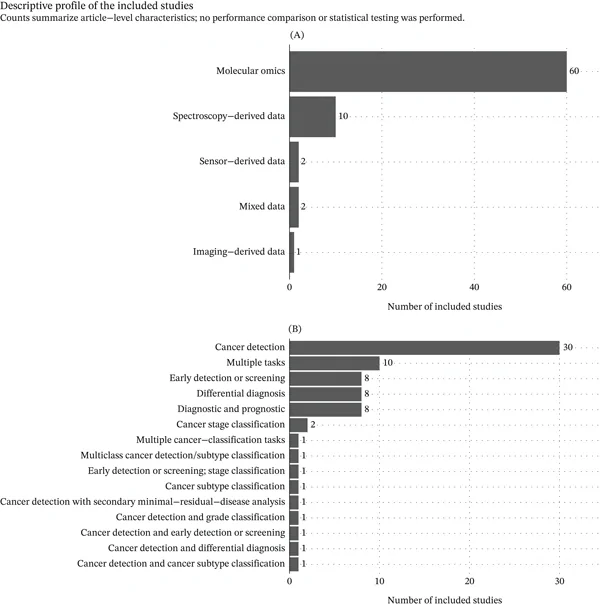
Descriptive profile of the 75 included studies. A shows the article‐level data family, classified as molecular omics or omics‐adjacent data. B shows the clinical application reported by each source article. Counts describe the evidence base only; no performance comparison or statistical testing was conducted.

**Table 1:** Article‐level selection counts and summary characteristics of the included evidence base.

Characteristic	Number of studies or entries
Reports assessed for eligibility	96
Reports excluded after revised eligibility assessment	21
Studies included in the review	75
Molecular omics studies	60
Omics‐adjacent studies	15
True multiomics studies	2
Internal validation only	60
Internal and external validation	8
External validation only	4
No validation	2
Unclear validation	1
Studies contributing extractable SVM performance	72
Article‐level SVM performance entries	544
High overall PROBAST risk of bias	72
Unclear overall PROBAST risk of bias	3

### 3.3. Validation Strategies

Sixty studies used internal validation only, eight reported both internal and external validation, four used external validation only, two reported no validation, and one was unclear. Internal validation dominated every data family (Figure 3). This pattern limits claims about transportability and clinical implementation, even when internally reported performance was high.

**Figure 3:**
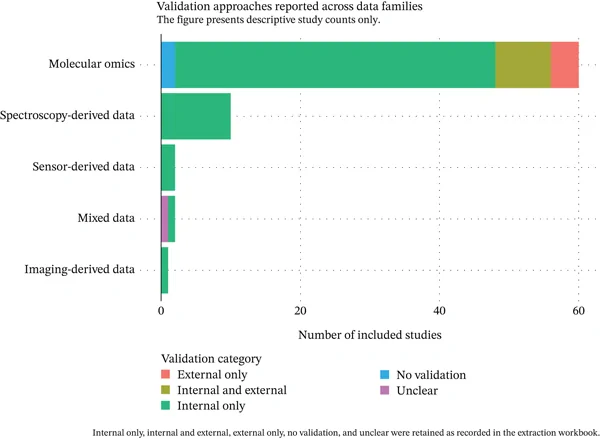
Validation approaches reported across data families. Bars present article‐level counts for internal‐only, internal‐and‐external, external‐only, no‐validation, and unclear categories. External validation required a cohort, institution, dataset, or source population independent of model development and feature selection.

### 3.4. Reported SVM Performance

Seventy‐two of the 75 included studies contributed at least one extractable numerical SVM‐specific result, yielding 544 article‐level performance entries. The remaining three studies (S037, S042, and S074) did not report a numerical SVM estimate that met the extraction criteria. Extracted measures included AUC, accuracy, sensitivity, specificity, predictive values, F1 score, precision, balanced accuracy, test error, and author‐defined metrics. We preserved each value as published; none was transformed, averaged, ranked, or pooled. Figure S2 shows metric availability by study. Reported results varied within and across clinical applications. For example, an externally evaluated urinary peptide classifier for solid tumors reported an AUC of 0.82, sensitivity of 0.69, and specificity of 0.81 [19]. A metabolomics SVM for esophageal squamous cell carcinoma reported test accuracy of 0.98 [20]. Multicenter urinary‐proteomic prostate studies reported externally evaluated discrimination, but clinical‐variable nomograms sometimes modestly improved performance over the SVM peptide score alone [21, 22]. These examples illustrate why individual results require interpretation within their own target population, reference standard, validation design, and comparator context. Several source articles reported AUC, accuracy, sensitivity, or specificity at or near 1.00. Such results were not treated as evidence of clinical readiness. For example, the imaging‐derived pancreatic ductal adenocarcinoma (PDAC) study reported very high internal performance in specific subgroups, but it remained a retrospective internally evaluated model [23]. Near‐perfect values in small, high‐dimensional datasets may reflect restricted case–control spectra, feature selection outside resampling, data leakage, or overfitting; these concerns were incorporated into PROBAST and the narrative interpretation.

### 3.5. Comparison With Alternative Models

Comparisons with alternative classifiers were heterogeneous and frequently incomplete. Some source articles reported SVM as the best performing method; some reported comparable performance, and others favored random forests, automated machine learning, neural networks, logistic models, nomograms, or hybrid models. For example, automated machine learning outperformed the standalone SVM in two metabolomics datasets [24], whereas SVM was one of several models with similar discrimination in a cell‐free DNA (cfDNA) methylation study [3]. The review therefore does not support a general claim that SVM is superior. Study‐level comparator details are provided in Table S2 and File S1.

### 3.6. Risk of Bias

In the separate PROBAST assessment, 72 studies were judged to be at high overall risk of bias and three (S008, S042, and S054) were rated unclear; none were rated low overall. The overall high‐risk PROBAST count (72) and the performance‐reporting denominator (72) are numerically equal but refer to different, only partially overlapping study sets. S042 belonged to both groups because it lacked an extractable SVM‐specific numerical result and received an unclear overall PROBAST judgment; this overlap does not make the two 72‐study sets equivalent. The complete domain‐level distribution was as follows: Participant selection was high risk in 65 studies and unclear in 10; predictors were low risk in two, unclear in 68, and high risk in five; outcome was low risk in 47 and unclear in 28; and analysis was high risk in 72 and unclear in three. High analysis‐domain risk most often reflected small effective sample sizes, feature selection or tuning that was not adequately nested, limited calibration, or absent independent validation (Figure 4; Figure S1).

**Figure 4:**
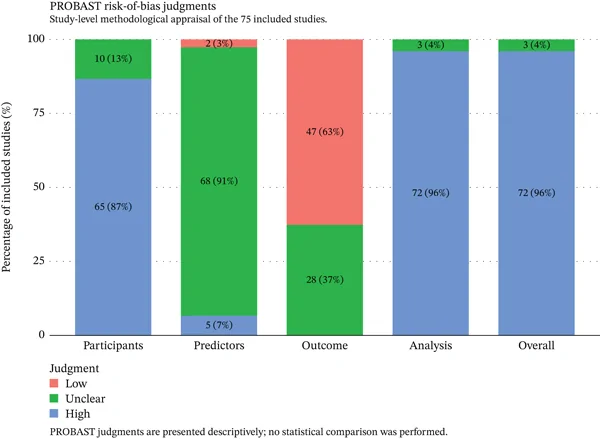
PROBAST risk‐of‐bias judgments for the 75 included studies. Stacked bars show the percentage and number of studies rated low, unclear, or high in each domain and overall. Judgments are presented descriptively and were used to qualify the narrative conclusions; no statistical comparison was performed.

## 4. Discussion

### 4.1. Principal Findings

This review maps a broad and methodologically diverse literature rather than estimating a single SVM performance value. Most studies used a single molecular omics layer, only two met the strict definition of multiomics integration, and a separate group used spectroscopy, sensors, imaging, or mixed inputs. Cancer detection predominated, but prognostic, staging, subtype, and treatment‐related models were also present. These tasks are not interchangeable and should not be represented by a common diagnostic summary. The evidence was dominated by internal validation. Only 12 studies reported any external validation, and only four used external validation without also relying on internal development results. External validation itself varied in independence and size, so its presence should not be interpreted as sufficient evidence of clinical utility [15–17].

### 4.2. Interpretation of High Reported Performance

Near‐perfect results were common enough to require critical interpretation, but not formal sensitivity analysis because no pooled analysis was undertaken. Very high performance can be genuine in a narrowly defined dataset, yet it can also arise from small samples, strong case–control separation, duplicate or correlated observations, nonnested feature selection, repeated use of the test data, or flexible threshold selection. The predominance of high analysis‐domain PROBAST judgments supports cautious interpretation. Prospective evaluation, calibration, locked preprocessing, and independent multi‐institutional testing are needed before clinical claims.

### 4.3. SVM in Relation to Alternative Algorithms

The rationale for reviewing SVM was its repeated use in high‐dimensional cancer studies and its role as a benchmark model, not an assumption of superiority. The study‐level comparisons were mixed. In some datasets SVM was competitive or best; in others, automated machine learning, random forests, neural networks, or combined clinical models performed better. This variability indicates that algorithm choice should follow the target question, sample size, feature structure, interpretability requirements, and validation plan rather than model reputation alone.

### 4.4. Data Modality and Reproducibility

Molecular layers differed in specimen handling, preprocessing, feature stability, and public‐data availability. Proteomic models may be particularly sensitive to laboratory and platform variation, whereas transcriptomic studies often benefit from larger public datasets; however, the present review did not statistically compare modalities. Reproducibility would be strengthened by locked pipelines, external cohort testing, calibration reporting, code and data sharing, and adherence to Findable, Accessible, Interoperable, and Reusable (FAIR) principles [25, 26].

### 4.5. Geographic Representation, Fairness, and Regulation

Forty‐two articles had a first author whose first‐listed institutional affiliation was in China. That bibliometric concentration was distinct from the geography of the evidence itself: 35 studies involved Chinese participants, biospecimens, local development or validation cohorts, or China‐linked source datasets. Demographic subgroup reporting was sparse. Neither pattern establishes bias on its own, yet transportability remains uncertain when models are not evaluated across ancestry, healthcare systems, disease prevalence, specimen workflows, and measurement platforms. Medical AI trained on skewed data may reproduce or amplify inequities embedded in healthcare data [27]. In the included literature, calibration, clinical‐utility assessment, subgroup fairness, data‐governance procedures, and regulatory pathways were rarely or inconsistently reported. We therefore did not treat country as a causal exposure or undertake an unsupported crosscountry effectiveness comparison. Future studies should prespecify subgroup analyses, document missingness and data provenance, test calibration across settings, and define privacy, consent, cybersecurity, and postdeployment monitoring procedures.

### 4.6. Implications for Future Research

The heterogeneity that prevents an overall meta‐analysis also identifies useful directions for focused reviews. The current evidence map can support future studies restricted to one cancer, one clinical task, one specimen and platform, comparable controls, and a consistent validation framework. For example, cancer‐detection studies with independently validated AUC, sensitivity, and specificity could be examined separately from prognostic or subtype‐classification studies. Such narrower questions may permit reliable quantitative synthesis and clinically interpretable conclusions.

### 4.7. Limitations

The principal limitation is substantial clinical, methodological, and statistical heterogeneity. The included studies differed in cancer, task, population, controls, specimen, platform, preprocessing, feature selection, SVM configuration, validation, thresholding, and reported outcomes. Pooling them would create a summary estimate without a coherent clinical meaning; therefore, this review used structured narrative reporting only. This review summarizes evidence identified up to August 4, 2024—the prespecified search end point for the evidence base evaluated during manuscript preparation and submission. Reports published after that date were outside the review period and may alter the observed distribution of methods, validation practices, and clinical applications. Even so, the present synthesis offers a structured foundation for future updates as new studies emerge. The search was restricted to English‐language reports, so language bias remains possible. Google Scholar was queried with a reproducible but restrictive all‐title strategy that returned 14 records and may have reduced sensitivity. A chance‐corrected interreviewer agreement statistic was not prespecified or calculated, which limits quantitative assessment of screening consistency even though every record was assessed by at least two reviewers, disagreements were discussed, and unresolved cases were adjudicated by Armin Zarinkhat as the subject‐matter expert. Calibration, clinical utility, demographic fairness, and prospective deployment were rarely reported. Risk‐of‐bias judgments were therefore used to qualify the narrative synthesis; without a pooled outcome and with high overall risk in almost all studies, a formal risk‐of‐bias sensitivity or subgroup analysis was not appropriate.

## 5. Conclusion

SVM models have been evaluated across many cancer‐related molecular omics and omics‐adjacent applications, but the literature is dominated by single‐modality studies, internal validation, heterogeneous outcomes, and high risk of bias. The evidence does not justify a universal estimate of SVM accuracy, claims of multiomics superiority, or routine clinical implementation. This review provides an article‐level evidence map that can pave the way for cancer‐specific, modality‐specific, and externally validated investigations, including future quantitative reviews of sufficiently homogeneous detection questions.

## Author Contributions

Zhina Mohamadi, Erfan Abtahi, Zahra Sadat Shayegh, Amin Fakhar, Amir Zinatshoar, Mahdi Biglari, and Fatemeh Rezaei contributed to study screening. Mehrafrin Ataei Kachouei, Mohammad Mahdi Shirani, Mohammadhosein Malekian, Amir Zinatshoar, Mahdi Biglari, Rozhina Mohammadi, and Fatemeh Rezaei contributed to data extraction. Erfan Abtahi, Mehrafrin Ataei Kachouei, Mohammad Mahdi Shirani, and Zahra Sadat Shayegh conducted the PROBAST assessments. Screening disagreements were first addressed through discussion; unresolved cases were adjudicated by Armin Zarinkhat as the subject‐matter expert. Zhina Mohamadi, Erfan Abtahi, Zahra Sadat Shayegh, and Amin Fakhar drafted the original manuscript. Rozhina Mohammadi led and coordinated the comprehensive revision of the work, including the article‐level reaudit and reconciliation of extracted data, verification of numerical and crossfile consistency, restructuring and substantive rewriting of the manuscript, preparation and coordination of the point‐by‐point responses, and assembly of the final submission package. Armin Zarinkhat supervised the work, supported figure preparation, and served as the corresponding author. Zhina Mohamadi, Rozhina Mohammadi, and Erfan Abtahi have contributed to the work equally and should be regarded as cofirst authors.

## Funding

No funding was received for this manuscript.

## Disclosure

All authors reviewed and approved the final manuscript.

## Ethics Statement

Not applicable. This review used published aggregate information and did not enroll human participants or animals.

## Consent

Not applicable

## Conflicts of Interest

The authors declare no conflicts of interest.

## Supporting Information

Additional supporting information can be found online in the Supporting Information section.

## Supporting information


**Supporting Information 1** Table S1: Database‐specific search strategies. Table S2: Article‐level characteristics of the 75 included studies.

**Supporting Information 2** Figure S1: Study‐level PROBAST judgments.

**Supporting Information 3** Figure S2: Reported SVM metric availability.

**Supporting Information 4** File S1: Systematic review—article‐level reporting workbook.

**Supporting Information 5** File S2: Completed PRISMA 2020 checklist.

## Data Availability

This study is a systematic review and does not involve the generation of new datasets. All data analyzed in this study were obtained from previously published sources. The extracted data from studies included in this systematic review are available as a supplementary file due to their large size. Citations for all referenced included studies are listed in the main reference section of the manuscript.
