# Supplementary material for: Support Vector Machine Models for Cancer Detection and Related Clinical Applications Using Omics and Omics‐Adjacent Data: A Systematic Review
Source: Biomed Res Int. 2026 Sep 29;2026:5935532. doi: 10.1155/bmri/5935532 (PMC13620843; doi:10.1155/bmri/5935532)
Supplement: Supplementary file 1 [file BMRI-2026-5935532-s001.pdf]

Supplementary Material: Support Vector Machine Models for Cancer Detection and Related Clinical Applications Using Omics and Omics-Adjacent Data

Zhina Mohamadi et al.

Supplementary Table S1. Database-specific search strategies

All database searches were completed through 4 August 2024.

Database	Search strategy	Records
PUBMED/MEDLINE	(("support vector machine"[Title/Abstract]) OR ("svm"[Title/Abstract])) OR ("Support Vector Machine"[Mesh]) AND ((((((("Neoplasms"[Title/Abstract]) OR ("Neoplasms"[Mesh])) OR ("carcinoma"[Title/Abstract])) OR ("carcinomas"[Title/Abstract])) OR ("Carcinoma"[Mesh])) OR ("sarcoma"[Title/Abstract])) OR ("sarcomas"[Title/Abstract])) OR ("Sarcoma"[Mesh]) AND (((((((((((((((((((((((((((((((((((((("Metabolomics"[Title/Abstract]) OR ("metabolic profiling"[Title/Abstract])) OR ("metabolic fingerprinting"[Title/Abstract])) OR ("metabolic phenotyping"[Title/Abstract])) OR ("metabolome analysis"[Title/Abstract])) OR (epigenetic[Title/Abstract])) OR (epigenetics[Title/Abstract])) OR (epigenomics[Title/Abstract])) OR ("epigenetic profiling"[Title/Abstract])) OR ("epigenetic analysis"[Title/Abstract])) OR ("epigenetic modifications"[Title/Abstract])) OR ("epigenetic mechanisms"[Title/Abstract])) OR (proteomics[Title/Abstract])) OR ("protein analysis"[Title/Abstract])) OR ("protein profiling"[Title/Abstract])) OR ("Proteome research"[Title/Abstract])) OR ("proteome analysis"[Title/Abstract])) OR ("transcriptomics"[Title/Abstract])) OR ("gene expression profiling"[Title/Abstract])) OR ("RNA sequencing"[Title/Abstract])) OR ("RNA-seq"[Title/Abstract])) OR ("transcriptome profiling"[Title/Abstract])) OR (genomics[Title/Abstract])) OR ("genome sequencing"[Title/Abstract])) OR ("genetic analysis"[Title/Abstract])) OR ("genome mapping"[Title/Abstract])) OR ("genomic profiling"[Title/Abstract])) OR ("genetic profiling"[Title/Abstract])) OR (Biomarker[Title/Abstract])) OR (biomarkers[Title/Abstract])) OR (gene[Title/Abstract])) OR (NGS[Title/Abstract])) OR ("Next generation sequencing"[Title/Abstract])) OR ("Micro RNA"[Title/Abstract])) OR (miRNA[Title/Abstract])) OR (protein[Title/Abstract])) OR ("Metabolomics"[Mesh])) OR ("Epigenomics"[Mesh])) OR ("Proteomics"[Mesh])) OR ("Gene Expression Profiling"[Mesh])	677

Database	Search strategy	Records
WEB OF SCIENCE	(TS=(Metabolomics) OR TS=(metabolic profiling) OR TS=(metabolic fingerprinting) OR TS=(metabolic phenotyping) OR TS=(metabolome analysis) OR TS=(epigenetic) OR TS=(epigenetics) OR TS=(epigenomics) OR TS= ("epigenetic profiling") OR TS=("epigenetic analysis") OR TS=("epigenetic modifications") OR TS=("epigenetic mechanisms") OR TS=(proteomics) OR TS=("protein analysis") OR TS=("protein profiling") OR TS=("Proteome research") OR TS=("proteome analysis") OR TS=("transcriptomics") OR TS=("gene expression profiling") OR TS=("RNA sequencing") OR TS=("RNA-seq") OR TS=("transcriptome profiling") OR TS=(genomics) OR TS=("genome sequencing") OR TS=("genetic analysis") OR TS= ("genome mapping") OR TS=("genomic profiling") OR TS=("genetic profiling") OR TS= (Biomarker) OR TS=(biomarkers) OR TS=(gene) OR TS=(NGS) OR TS=("Next generation sequencing") OR TS= ("Micro RNA") OR TS=(miRNA) OR TS=(protein) OR TS=(genotype) OR TS=(phenotype)) AND (TS=(Support vector machine) OR TS=(SVM)) AND (TS=(Neoplasm) OR TS=(Neoplasms) OR TS=("carcinoma") OR TS=("carcinomas") OR TS=("sarcoma") OR TS=("sarcomas") OR TS=(malignant) OR TS=(malignancy) OR TS=(malignancies)) AND (TS=(Detection) OR TS=(diagnosis) OR TS=(diagnostic) OR TS= (detector) OR TS=(discovery))	642
SCOPUS	(support vector machine OR SVM) AND (Cancer OR Cancers OR tumor OR tumors OR malignancy OR malignancies OR Malignant OR carcinoma OR carcinomas OR sarcoma OR sarcomas OR neoplasm OR neoplasms OR "malignant growth") AND (metabolomics OR "metabolic profiling" OR "metabolic fingerprinting" OR "metabolic phenotyping" OR "metabolome analysis" OR epigenomics OR epigenetics OR "epigenetic profiling" OR "epigenetic analysis" OR "epigenetic modifications" OR "epigenetic mechanisms" OR proteomics OR "protein analysis" OR "protein profiling" OR "proteome research" OR "proteome analysis" OR "transcriptomics" OR "gene expression profiling" OR "transcriptome analysis" OR "RNA sequencing" OR "RNA-seq" OR "transcriptome profiling" OR genomics OR "genome sequencing" OR "genetic analysis" OR "genome mapping" OR "genomic profiling" OR "genetic profiling" OR Biomarker OR biomarkers OR gene OR NGS OR "Next generation sequencing" OR "Micro RNA" OR miRNA OR protein OR genotype OR phenotype) AND (Detection OR Diagnosis OR Diagnostic OR Detector OR Discovery)	2149

Database	Search strategy	Records
GOOGLE SCHOLAR	`allintitle: "support vector machine" "metabolomics" OR "phenotype" OR "genotype" OR "metabolic profiling" OR "metabolic fingerprinting" OR "metabolic phenotyping" OR "metabolome analysis" OR "epigenetic" OR "epigenetics" OR "epigenomics" OR "epigenetic profiling" OR "epigenetic analysis" OR "epigenetic modifications" OR "epigenetic mechanisms" OR "proteomics" OR "protein analysis" OR "protein profiling" OR "Proteome research" OR "proteome analysis" OR "transcriptomics" OR "gene expression profiling" OR "RNA sequencing" OR "RNA-seq" OR "transcriptome profiling" OR "genomics" OR "genome sequencing" OR "genetic analysis" OR "genome mapping" OR "genomic profiling" OR "genetic profiling" OR "Biomarker" OR "biomarkers" OR "gene" OR "NGS" OR "Next generation sequencing" OR "Micro RNA" OR "miRNA" OR "protein" "cancer"`	14
Total		3482

Supplementary Table S2. Article-level characteristics of the 75 included studies

ID	Year	First-author country	Study-data geography	Cancer	Clinical task	Data family	Omics layer	Validation	Key reported SVM performance	SVM versus alternatives	ROB	DOI
S001	2022	United States	United States	Lung cancer	Cancer detection	Molecular omics	Epigenomics	Internal only	AUC=1.00 (0.90-1.00); sensitivity=0.94; specificity=1.00	SVM tied on AUC	High	10.3389/fonc.2022.790645
S002	2018	Germany	Germany/European source cohorts	Five solid-tumor types	Cancer detection	Molecular omics	Proteomics	External only	AUC=0.82 (0.79-0.85); sensitivity=0.69; specificity=0.81	Clinical-variable nomogram slightly improved AUC	High	10.1038/s41598-018-23585-y
S003	2005	Italy	Dataset origins NR	Colon adenocarcinoma; medulloblastoma	Diagnostic and prognostic	Molecular omics	Transcriptomics	Internal only	Single SVM test error 17.74% +/- 10.87%; ensemble better but exact value NR	Ensemble better than single SVM	High	NR
S004	2024	United States	RCC NR; OC South Korea	Renal cell carcinoma; ovarian cancer	Multiple tasks	Molecular omics	Metabolomics	Internal only	RCC AUC 0.93; OC AUC 0.82	Alternative model performed better	High	10.1021/jasms.3c00403
S005	2007	Brazil	Brazil	Hodgkin lymphoma	Cancer detection	Molecular omics	Proteomics	Internal only	Accuracy, sensitivity, and specificity all 1.00 in LOO	No alternative model comparison	High	NR
S006	2014	United States	United States	Colon cancer	Cancer detection	Molecular omics	Epigenomics	Internal only	Test accuracy 1.00; training sensitivity 0.96, specificity 1.00	No alternative classifier comparison	High	10.1158/1940-6207.CAPR-13-0407
S008	2023	United States	United States	Pancreatic ductal adenocarcinoma	Early detection or screening	Imaging-derived data	Not conventional omics	Internal only	Non-CP PCA/Gaussian AUC 0.988; CP AUC 1.00	No alternative classifier comparison	Unclear	10.14309/ctg.0000000000000548
S009	2021	China	China	Esophageal squamous cell carcinoma	Cancer detection	Molecular omics	Metabolomics	Internal only	AUC 0.979; accuracy 0.980; sensitivity 0.960; specificity 1.000	SVM performed best on accuracy	High	10.1016/j.jpha.2020.11.009
S010	2019	Taiwan	Taiwan	Endometrial cancer	Cancer detection	Molecular omics	Metabolomics	Internal only	Test accuracy 0.78; sensitivity 0.75; specificity 0.80; test AUC NR	SVM comparable	High	10.1007/s11306-019-1609-z
S012	2018	China	China	Nasopharyngeal carcinoma	Cancer detection	Spectroscopy-derived data	Not conventional omics	Internal only	WPCA-SVM AUC=0.982; accuracy=0.9915; sensitivity=0.9879; specificity=0.9936	SVM variant performed best	High	10.1364/OE.26.028661
S013	2022	China	China	Colorectal cancer	Diagnostic and prognostic	Molecular omics	Transcriptomic and proteomic...	No validation	AUC=0.878 in GEO data; AUC=0.743 in protein data	SVM was not compared with alternatives	High	10.1266/ggs.21-00072
S014	2021	China	China	Colorectal cancer	Diagnostic and prognostic	Molecular omics	Proteomics	Internal only	AUC=0.933	No alternative classifier comparison	High	10.1080/15384101.2021.1903208
S015	2020	France	France/European multicenter cohorts	Peripheral T-cell lymphoma	Cancer subtype classification	Molecular omics	Transcriptomics	Internal only	Accuracy=140/153=0.915 for specified PTCL; inter-lab concordance=36/40=0.90	No alternative supervised classifier comparison	High	10.3324/haematol.2019.226647
S016	2012	China	China	Breast cancer	Cancer detection	Molecular omics	Proteomics	Internal only	Held-out accuracy=0.9178; sensitivity=0.9189; specificity=0.9167	SVM outperformed CA15-3 in the same held-out group	High	10.1186/1746-1596-7-45

ID	Year	First-author country	Study-data geography	Cancer	Clinical task	Data family	Omics layer	Validation	Key reported SVM performance	SVM versus alternatives	ROB	DOI
S017	2010	China	China	Breast cancer	Cancer detection	Molecular omics	Proteomics	Internal only	Blind-test AUC=0.972; sensitivity=0.9645; specificity=0.9487; PPV=0.960	No alternative classifier comparison	High	10.1007/s00432-010-0775-1
S018	2023	Germany	Spain and Austria	Prostate cancer	Cancer detection	Molecular omics	Proteomics	Internal and external	First validation AUC=0.81 (0.77-0.85), sensitivity=0.93, specificity=0.69; external-site sensitivity=0.837	SVM outperformed PSA/PSAD/ERSPC; nomogram modestly improved AUC	High	10.3390/cancers15041166
S019	2022	Germany	Austria	Prostate cancer	Cancer stage classification	Molecular omics	Proteomics	External only	AUC=0.803 (0.73-0.86); sensitivity=0.875; specificity=0.646; NPV=0.918; PPV=0.550	Nomograms had slightly higher AUC; differences versus 19-BM were most...	High	10.1007/s00345-022-04077-1
S020	2008	Germany	Germany	Breast cancer	Cancer detection	Molecular omics	Metabolomics	Internal only	31-feature sensitivity=0.8767, specificity=0.8990; nested-RFE sensitivity=0.8939, specificity=0.8889	No supervised alternative model comparison	High	10.1080/13547500802012858
S021	2015	United States	United States and Canada	Early-stage ovarian cancer	Early detection or screening	Molecular omics	Metabolomics	Internal only	Accuracy=1.00; sensitivity=1.00; specificity=1.00 for 16-metabolite panel under LOOCV	No fair alternative classifier comparison	High	10.1038/srep16351
S023	2017	Germany	Germany and Italy	PDAC and other pancreatico-biliary malignancies	Differential diagnosis	Molecular omics	Transcriptomics, mRNA and miRNA	Internal only	Accuracy=0.930; sensitivity=0.917; specificity=0.945 for PDAC vs chronic pancreatitis	Combined model outperformed miRNA-only SVM	High	10.18632/oncotarget.22601
S025	2006	United States	United States	Ovarian and prostate cancer	Early detection or screening	Molecular omics	Proteomics	Internal only	Ovarian linear SVM accuracy=0.97, sensitivity=0.99, specificity=0.92; prostate polynomial SVM accuracy=0.82, sen...	Kernel comparison only	High	NR
S027	2021	Argentina	Argentina	Clear cell renal cell carcinoma	Early detection or screening; stage cla...	Molecular omics	Lipidomics	Internal only	Test AUC=0.90, accuracy=0.771, sensitivity=0.75, specificity=1.00 for 16-lipid panel	No direct alternative classifier comparison	High	10.1021/acs.jproteome.0c00663
S028	2023	India	Public TCGA and GEO datasets	Colorectal cancer	Early detection or screening	Molecular omics	Transcriptomics	No validation	TCGA SVM accuracy=1.00; GSE50760 SVM accuracy=0.934; validation level unclear/apparent	Alternative external model performed better/was the only externally e...	High	10.3390/genes14101836
S029	2009	South Korea	Korea; public tissue datasets	Breast cancer	Early detection or screening	Molecular omics	Metabolomics in final model;...	Internal only	SVM test AUC=0.79; approximate accuracy=0.70, sensitivity=0.80, specificity=0.60	SVM was comparable with random forest	High	10.1093/bioinformatics/btp558
S030	2008	China	China	Non-small-cell lung cancer	Cancer detection	Molecular omics	Proteomics	Internal only	Accuracy=0.9375; sensitivity=0.875; specificity=1.00	No alternative classifier comparison	High	10.1007/s00408-008-9093-7

ID	Year	First-author country	Study-data geography	Cancer	Clinical task	Data family	Omics layer	Validation	Key reported SVM performance	SVM versus alternatives	ROB	DOI
S031	2022	Latvia	Latvia	Gastric adenocarcinoma	Cancer detection	Sensor-derived data	Not conventional omics	Internal only	Cluster DTWARP-complete linkage with InfoGain: AUC=0.716; accuracy=0.7487; sensitivity=0.6073; specificity=0.8248	Alternative model performed better	High	10.3390/diagnostics12020491
S032	2022	China	China	Bladder cancer	Cancer detection	Molecular omics	Transcriptomics	Internal only	AUC=0.875 (95% CI 0.799-0.942); accuracy=0.816; sensitivity=0.800; specificity=0.814 under LOOCV	SVM was not directly compared	High	10.1039/d2mo00107a
S033	2022	China	China	Papillary thyroid cancer	Diagnostic and prognostic	Molecular omics	Glycomics	Internal only	Accuracy=0.92 [internal testing set]	SVM performed best	High	10.1016/j.ijbiomac.2022.06.102
S034	2021	China	China	Thyroid neoplasm; papillary thyroid cancer	Cancer detection and cancer subtype cla...	Molecular omics	Transcriptomics	Internal only	Binary validation: AUC=0.998 and accuracy=0.970 in one split; mean accuracy=0.965 over 1000 repeated 60/40 splits	SVM was not directly compared	High	10.1007/s12020-020-02523-x
S035	2022	China	China	Early-stage hepatocellular carcinoma	Multiple tasks	Molecular omics	Immunomics	Internal only	AdaBoost-SVM apparent performance on 47 diagnostic subjects: AUC=0.992; accuracy=0.915; sensitivity=0.929; speci...	SVM-based model outperformed the AFP comparator	High	10.1136/jitc-2021-003133
S036	2022	China	Public TCGA dataset	Colon adenocarcinoma	Multiple tasks	Molecular omics	Transcriptomics	Internal only	Diagnosis: AUC=0.9967; accuracy=0.9846; sensitivity=0.9692; specificity=0.9894; precision=0.9450; F1=0.9529	Alternative model performed better	High	10.1016/j.compbiomed.2022.105409
S037	2023	China	China	Esophageal squamous cell carcinoma	Cancer detection	Molecular omics	Proteomics	Internal only	NR	Alternative model performed better	High	10.1016/j.clon.2023.06.014
S038	2024	China	China	Liver cancer	Cancer stage classification	Spectroscopy-derived data	Not conventional omics	Internal only	PLS-SVM accuracy=0.875 [unknown internal testing set]	Insufficient information	High	NR
S039	2013	France	France	Hepatocellular carcinoma	Differential diagnosis	Spectroscopy-derived data	Not conventional omics	Internal only	Freeze-dried serum using all spectra: accuracy=0.9155; sensitivity=0.9459; specificity=0.8824; AUC NR	SVM was not directly compared with another predictive classifier	High	10.1039/c3an00245d
S040	2023	China	Public international GEO datasets	Twelve cancer types	Cancer detection and early detection or...	Molecular omics	Transcriptomics	Internal only	Held-out test: AUC=0.934 (95% CI 0.927-0.941); accuracy=0.870; sensitivity=0.828; specificity=0.896	SVM was not directly compared	High	10.3389/fendo.2023.1099703
S041	2022	China	China	Hepatocellular carcinoma	Differential diagnosis	Molecular omics	Glycomics	Internal only	Validation SVM-HCC: AUC=0.857 (95% CI 0.769-0.944); accuracy=0.812; sensitivity=0.846; specificity=0.797	Alternative model performed better	High	10.1515/cclm-2022-0715
S042	2024	Canada	Canada	Early-stage high-grade serous ovarian carcinoma	Early detection or screening	Mixed data	Not conventional omics	Unclear	NR	Insufficient information	Unclear	10.1021/acssensors.3c01995

ID	Year	First-author country	Study-data geography	Cancer	Clinical task	Data family	Omics layer	Validation	Key reported SVM performance	SVM versus alternatives	ROB	DOI
S043	2022	China	Public TCGA dataset	Colorectal cancer	Diagnostic and prognostic	Molecular omics	Transcriptomics	Internal only	Held-out same-TCGA test AUC=0.9956; training cross-validation accuracy=0.9685	SVM was not directly compared with another diagnostic classifier	High	10.1186/s13062-022-00342-w
S045	2014	China	China	Hepatocellular carcinoma	Differential diagnosis	Molecular omics	Proteomics	Internal only	Held-out test: AUC=0.751; accuracy=0.757; sensitivity=0.825; specificity=0.676	Alternative model performed better	High	10.1111/jgh.12545
S046	2016	China	China	Thyroid cancer and thyroid nodules	Differential diagnosis	Molecular omics	Transcriptomics	External only	Validation: accuracy=0.917; sensitivity=0.927; specificity=0.901; PPV=0.934; NPV=0.890	SVM was not directly compared	High	10.1186/s13046-016-0447-3
S048	2022	China	China	Bladder cancer	Multiple tasks	Sensor-derived data	Not conventional omics	Internal only	Held-out test: accuracy=0.9667; sensitivity=1.000; specificity=0.8333	SVM was not directly compared	High	10.1021/acssensors.2c00467
S050	2020	Turkey	Public datasets; original countries mixed/NR	Ovarian cancer; leukemia; central nervous sys...	Multiple cancer-classification tasks	Molecular omics	Transcriptomics	Internal only	ReliefF-SVM mean accuracy: ovarian=0.9747; leukemia=0.9806; CNS=0.8072	Alternative model performed better	High	10.1016/j.mehy.2020.109577
S052	2021	Iran	Iran with public discovery datasets	Colorectal cancer	Cancer detection	Molecular omics	Epigenomics	Internal only	Held-out test: sensitivity=0.98; specificity=0.99; accuracy=0.98; AUC NR. Full-cohort ROC AUC=0.98	SVM was not directly compared	High	10.1016/j.cancergen.2020.12.007
S053	2023	China	China	Gastric and colorectal cancer	Multiple tasks	Spectroscopy-derived data	Not conventional omics	Internal only	Cancer detection accuracy=1.00 for gastric versus healthy and colorectal versus healthy; AUC/sensitivity/specifi...	SVM was comparable for detection; alternative model better for some s...	High	10.1039/d3an01259j
S054	2015	China	Public international GEO datasets	Lung adenocarcinoma	Cancer detection	Molecular omics	Transcriptomics	External only	External accuracies: 0.9848; 0.9167; 0.9762; 0.9800; 0.9675; 0.9167 across six datasets	SVM was not directly compared	Unclear	10.1016/j.gene.2015.03.008
S055	2010	China	China	Acute leukemia	Cancer detection with secondary minimal...	Molecular omics	Proteomics	Internal only	Held-out validation: AUC=0.998 (95% CI 0.994-1.002); sensitivity=0.97; specificity=1.00	SVM was not directly compared	High	10.1002/pmic.200900513
S056	2023	Iran	Public TCGA and GEO datasets	Stomach adenocarcinoma	Diagnostic and prognostic	Molecular omics	Epigenomics	Internal and external	External GSE30601: AUC=0.97; F1=0.93; precision=0.94; specificity=0.86; sensitivity=0.93; accuracy=0.91	SVM performed best	High	10.1111/jcmm.17693
S057	2015	South Korea	South Korea; independent public GEO datasets	Pancreatic ductal adenocarcinoma	Cancer detection	Molecular omics	Multi-omics	Internal and external	No single prespecified final model; representative miR-148a AUC=0.956 and BA=0.927 in discovery, external BA up...	SVM was not directly compared	High	10.1186/1471-2164-16-S9-S4
S058	2013	China	China; public GEO datasets	Prostate cancer	Cancer detection	Molecular omics	Transcriptomics	Internal only	Test AUC/accuracy: GSE6099 0.90/0.8907; GSE6956 0.89/0.8588; GSE21034 0.93/0.9271	SVM was not directly compared	High	10.1371/journal.pone.0063941

ID	Year	First-author country	Study-data geography	Cancer	Clinical task	Data family	Omics layer	Validation	Key reported SVM performance	SVM versus alternatives	ROB	DOI
S059	2013	United Kingdom	United Kingdom	Glioblastoma multiforme	Cancer detection	Spectroscopy-derived data	Not conventional omics	Internal only	Whole-serum patient test sensitivity=0.875; specificity=1.000	SVM was not directly compared	High	10.1007/s00216-013-7163-z
S061	2023	China	China	Non-small cell lung cancer	Multiple tasks	Molecular omics	Multi-omics	Internal and external	SVM component AUCs: Bincount 0.759; motif 0.815; protein marker 0.700. Hybrid LC score AUC=0.912	Hybrid integrated model performed best	High	10.1016/j.ebiom.2023.104553
S062	2021	China	China	AFP-negative hepatocellular carcinoma	Differential diagnosis	Molecular omics	Glycomics	Internal only	AUC=0.610; sensitivity=0.9608; specificity=0.2889 in training CV	Alternative model performed better	High	10.1002/ijc.33564
S063	2012	United States	United States	Non-small cell lung cancer	Multiple tasks	Molecular omics	Proteomics	Internal only	Three-class accuracy=0.94; NSCLC versus normal sensitivity=0.99 and specificity=0.95	SVM was not directly compared	High	NR
S064	2019	Spain	Spain	Colorectal cancer and advanced adenoma	Early detection or screening	Molecular omics	Transcriptomics	Internal only	Cross-validated advanced-neoplasm AUC=0.79 (0.723-0.841)	SVM was not directly compared	High	10.14309/ctg.0000000000000003
S065	2021	China	China	Breast cancer	Cancer detection	Molecular omics	Transcriptomics	Internal and external	Training LOOCV AUC=0.83; validation AUC=0.80	SVM performed best	High	10.3389/fonc.2021.752651
S067	2009	Germany	Germany	Breast cancer	Cancer detection	Molecular omics	Metabolomics	Internal only	10-fold sensitivity=0.835, specificity=0.906, MCC=0.743; LOO sensitivity=0.835, specificity=0.859	SVM was not directly compared on a locked test set	High	10.1186/1471-2407-9-104
S068	2010	China	China	Oral squamous cell carcinoma	Multiple tasks	Spectroscopy-derived data	Not conventional omics	Internal only	Blind calibration accuracy: SCC-normal 0.9524; SCC-OLK 0.9318; OLK-normal 0.7391	SVM was not directly compared	High	10.1002/jrs.2421
S069	2020	China	Public TCGA dataset; qRT-PCR cohort China	Head and neck squamous cell carcinoma	Diagnostic and prognostic	Molecular omics	Transcriptomics	Internal only	AUC=0.983; sensitivity=0.962; specificity=0.955	SVM was comparable	High	10.3233/CBM-190694
S070	2020	China	Public TCGA/GEO datasets; qRT-PCR cohort China	Stomach adenocarcinoma	Diagnostic and prognostic	Molecular omics	Transcriptomics	Internal only	AUC=0.983; sensitivity=0.971; specificity=0.969	SVM performed best	High	10.1002/mgg3.1512
S071	2020	United States	Public international datasets	Pancreatic ductal adenocarcinoma and precurso...	Multiple tasks	Molecular omics	Transcriptomics	Internal and external	Dataset AUCs 0.80-1.00; median near 0.95 across Table 2	SVM performed best	High	10.3389/fgene.2020.572284
S072	2023	Turkey	Turkey	Primary myelofibrosis	Cancer detection	Spectroscopy-derived data	Not conventional omics	Internal only	Accuracy=0.923-1.00 depending on FTIR range	SVM performed best	High	10.1016/j.bbagen.2023.130438
S073	2007	China	China	Esophageal squamous cell carcinoma	Early detection or screening	Molecular omics	Proteomics	Internal only	Accuracy=0.851; sensitivity=0.861; specificity=0.842; PPV=0.838 [LOOCV]	SVM was not directly compared	High	NR
S074	2019	China	Public GEO datasets	Pancreatic ductal adenocarcinoma	Cancer detection	Molecular omics	Transcriptomics	Internal and external	External accuracy approximately 0.80; exact SVM-specific value NR	Insufficient information	High	10.2217/bmm-2018-0273

ID	Year	First-author country	Study-data geography	Cancer	Clinical task	Data family	Omics layer	Validation	Key reported SVM performance	SVM versus alternatives	ROB	DOI
S075	2022	Thailand	Thailand	Cholangiocarcinoma	Cancer detection	Molecular omics	Proteomics	Internal only	Linear SVM All-7 AUC=0.961 (95% CI 0.885-1.000)	SVM performed best for All-7	High	10.3390/molecules27185904
S077	2021	China	China	Lung cancer	Cancer detection	Mixed data	Transcriptomics	Internal only	Advanced SVM AUC=0.903, sensitivity=0.900, specificity=0.732, PPV=0.851, NPV=0.811	SVM performed best among reported integrated variants	High	10.1002/ijc.33675
S078	2018	China	China	Colorectal cancer	Cancer detection	Molecular omics	Genomics	Internal only	Test AUC=0.8796; sensitivity=0.917; specificity=0.889; accuracy=0.900	SVM performed best	High	10.1159/000487571
S079	2017	China	Public ArrayExpress dataset	Gastric cancer	Cancer detection	Molecular omics	Transcriptomics	Internal only	Five-gene panel AUC=0.99; sensitivity=0.98; specificity=1.00; MCC=0.95	SVM was not directly compared with a different classifier	High	NR
S080	2021	China	China	Breast cancer stage classification	Multiple tasks	Spectroscopy-derived data	Not conventional omics	Internal only	Best oversampling GS-SVM accuracy=0.9545; best undersampling GA-SVM accuracy=1.00	SVM performed best	High	10.1016/j.pdpdt.2021.102199
S083	2004	China	China	Colorectal cancer	Differential diagnosis	Molecular omics	Proteomics	Internal only	Accuracy=0.867; sensitivity=0.89; specificity=0.83; PPV=0.89	SVM was not directly compared on the same task	High	10.3748/wjg.v10.i21.3127
S084	2005	China	China	Ovarian cancer	Cancer detection	Molecular omics	Proteomics	Internal only	Sensitivity=0.967; specificity=0.967; PPV=0.967; accuracy=0.967	SVM was not directly compared	High	10.1631/jzus.2005.B0227
S085	2020	China	China	Pancreatic ductal adenocarcinoma	Cancer detection and differential diagn...	Molecular omics	Transcriptomics	Internal and external	External PDAC vs CP+healthy: AUC=0.936 (95% CI 0.889-0.983); sensitivity=0.9368; specificity=0.9157; accuracy=0....	No alternative ML classifier; d-signature outperformed CA19-9 for PDA...	High	10.1136/gutjnl-2019-318860
S086	2020	China	Public GEO dataset; original cohort Netherlands	Breast, colorectal, NSCLC, glioblastoma, panc...	Multiclass cancer detection/subtype cla...	Molecular omics	Transcriptomics	Internal only	Data15 seven-class EPSVM alpha=0.1 mean NER=0.8133	EPSVM generally outperformed SVM-RFE+SVM and ReliefF+SVM	High	10.3390/s20051528
S091	2024	Singapore	Singapore	Breast cancer	Differential diagnosis	Spectroscopy-derived data	Not conventional omics	Internal only	Multimodal SVM AUC=0.9979 at 86/5 split; AUC=0.8571 at 61/30 split	SVM performed best for the multimodal dataset	High	10.1039/d3ra05723b
S094	2020	China	China	Hepatocellular carcinoma	Cancer detection	Molecular omics	Transcriptomics	Internal only	qPCR cohort: AUC=0.940 (95% CI 0.908-0.964); sensitivity=0.880; specificity=0.883	SVM signature outperformed AFP; no alternative ML comparison	High	10.21037/atm.2020.01.93
S096	2019	China	China	Glioma	Cancer detection and grade classification	Spectroscopy-derived data	Not conventional omics	Internal only	PC1+PC2+PC6 LOOCV: sensitivity=1.000; specificity=0.963; accuracy=0.996; AUC approximately 1.0	No alternative classifier	High	10.1117/1.JBO.24.9.095001

First-author country is the country of the first author's first-listed institutional affiliation. Study-data geography denotes the reported origin of participants, biospecimens, development cohorts, validation cohorts, or source datasets. These variables are not interchangeable. ROB, overall PROBAST risk of bias.

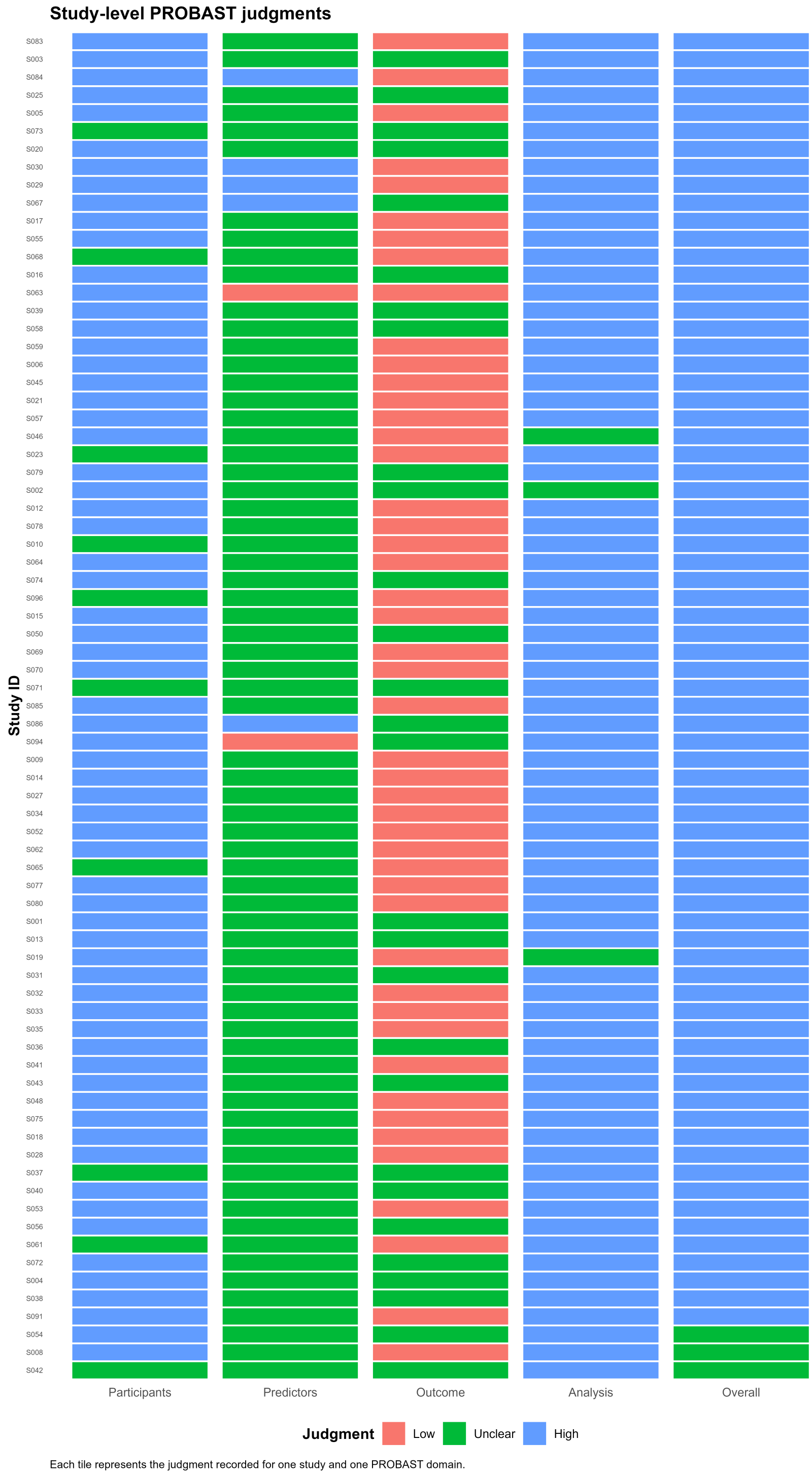

Supplementary Figure S1. Study-level PROBAST judgments. Each tile shows the recorded study-level PROBAST judgment for one domain or the overall assessment. Blue indicates high risk, green unclear risk, and red low risk.

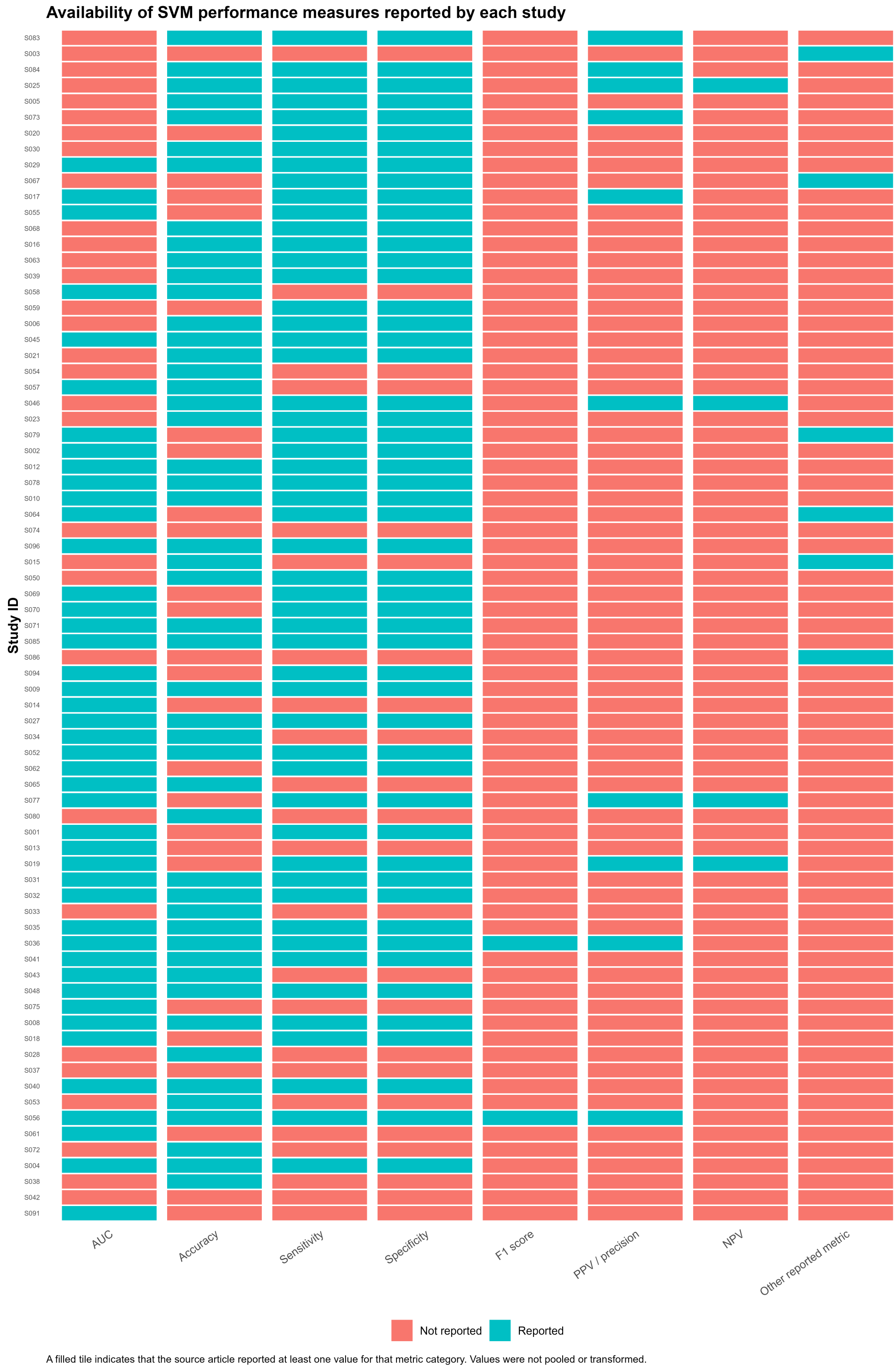

Supplementary Figure S2. Availability of reported SVM performance metrics. A filled tile indicates that the source article reported at least one value for the corresponding SVM metric category. Values were retained as reported and were not pooled or transformed.

Supplementary Data File 1

The accompanying Excel workbook contains the complete eligibility audit, article-level study characteristics, separate first-author affiliation and study-data geography fields, 544 reported SVM performance entries, and study-level PROBAST judgments. It is the authoritative structured data source for this revision.
