# Supplementary material for: Support Vector Machine Models for Cancer Detection and Related Clinical Applications Using Omics and Omics‐Adjacent Data: A Systematic Review
Source: Biomed Res Int. 2026 Sep 29;2026:5935532. doi: 10.1155/bmri/5935532 (PMC13620843; doi:10.1155/bmri/5935532)
Supplement: Supplementary file 2 [file BMRI-2026-5935532-s005.pdf]

Completed PRISMA 2020 Checklist

Review title: Support Vector Machine Models for Cancer Detection and Related Clinical Applications Using Omics and Omics-Adjacent Data: A Systematic Review

Section	Item	PRISMA 2020 reporting item	Location in the revised submission
Title	1	Identify the report as a systematic review.	Manuscript title.
Abstract	2	Report the main elements recommended for a systematic-review abstract.	Structured Abstract: Background, Methods, Results, and Conclusions.
Introduction	3	Describe the rationale for the review in the context of existing knowledge.	Introduction, paragraphs 1–4.
Introduction	4	Provide an explicit statement of the review objectives or questions.	Final paragraph of the Introduction.
Methods	5	Specify inclusion and exclusion criteria and how studies were grouped for synthesis.	Methods: Screening and eligibility; Synthesis approach.
Methods	6	Specify all information sources and the date each source was last searched.	Methods: Information sources and search strategy; Supplementary Table S1. Searches completed through 4 August 2024.
Methods	7	Present the full search strategies for all databases and other sources.	Supplementary Table S1.
Methods	8	Specify the study-selection process, including reviewer roles and any automation tools.	Methods: Screening and eligibility. Rayyan was used. A seven-reviewer team conducted screening, every record was independently assessed by at least two reviewers, and unresolved disagreements were adjudicated by Armin Zarinkhat as the subject-matter expert.
Methods	9	Specify the data-collection process, including the number of reviewers and procedures used to confirm data.	Methods: Data extraction and outcome organization. Seven reviewers contributed to structured data extraction, and entries were reconciled article by article during the final revision audit; Supplementary Data File 1 contains the resulting dataset.
Methods	10a	List and define all outcomes for which data were sought.	Methods: Data extraction and outcome organization; Reported SVM performance measures are listed in Results and the workbook.
Methods	10b	List and define other variables for which data were sought and describe assumptions about missing or unclear information.	Methods: Data extraction and outcome organization; unclear validation was retained when source reporting was insufficient.
Methods	11	Specify methods used to assess risk of bias in included studies.	Methods: Risk of bias. Four reviewers independently applied PROBAST; domain and overall judgments are reported in Figure 4, Supplementary Figure S1, and the workbook.
Methods	12	Specify the effect measure(s) used for each outcome.	No common review-level effect measure was imposed. Article-level AUC, accuracy, sensitivity, specificity, predictive values, F1 score, precision, balanced accuracy, error, and author-defined metrics were retained as reported.
Methods	13a	Describe processes used to decide which studies were eligible for each synthesis.	Methods: Screening and eligibility; Synthesis approach.
Methods	13b	Describe methods required to prepare data for presentation or synthesis.	Methods: Data extraction and outcome organization. Published values were preserved without transformation, averaging, ranking, or pooling.
Methods	13c	Describe methods used to tabulate or visually display results.	Methods: Synthesis approach; Figures 1–4; Supplementary Table S2; Supplementary Figures S1–S2.
Methods	13d	Describe methods used to synthesize results and justify the choice.	Methods: Synthesis approach. Structured narrative reporting and descriptive counts were used because tasks, populations, platforms, validation designs, thresholds, and metrics were not comparable.
Methods	13e	Describe methods used to explore possible causes of heterogeneity.	Heterogeneity was described by data family, clinical task, validation category, comparator context, and risk of bias; no formal subgroup test was performed.
Methods	13f	Describe sensitivity analyses used to assess robustness.	Not performed. The rationale is stated in Methods and Limitations: no pooled outcome was calculated and high overall risk of bias was nearly universal.
Methods	14	Describe methods used to assess risk of bias due to missing results.	No formal reporting-bias test was performed because no pooled synthesis was conducted; this is implicit in the narrative-only synthesis and acknowledged in Limitations.

Section	Item	PRISMA 2020 reporting item	Location in the revised submission
Methods	15	Describe methods used to assess certainty in the body of evidence.	No formal certainty-of-evidence framework was applied because the review did not estimate a common effect. PROBAST was used to qualify interpretation at study level.
Results	16a	Describe results of the search and selection process, ideally with a flow diagram.	Results: Study selection; Figure 1 reports all retained PRISMA-stage counts from 3482 identified records to 75 included studies.
Results	16b	Cite studies that appeared eligible but were excluded and explain why.	Supplementary Data File 1, Eligibility_Decisions sheet, retains all 21 exclusions and reasons.
Results	17	Cite each included study and present its characteristics.	Supplementary Table S2 and Supplementary Data File 1; study DOIs are linked where available.
Results	18	Present risk-of-bias assessments for each included study.	Results: Risk of bias; Figure 4; Supplementary Figure S1; Supplementary Data File 1.
Results	19	Present results for all outcomes for each study.	Supplementary Table S2 and the Reported_SVM_Performance sheet in Supplementary Data File 1.
Results	20a	Summarize characteristics and risk of bias of studies contributing to each synthesis.	Results: Study characteristics, validation strategies, reported performance, comparison with alternative models, and risk of bias.
Results	20b	Present results of statistical syntheses.	Not applicable; no meta-analysis, pooled estimate, median/IQR synthesis, or formal subgroup comparison was performed.
Results	20c	Present investigations of possible causes of heterogeneity.	Descriptive stratification is reported by data family, task, validation design, comparator context, and PROBAST; no inferential heterogeneity analysis was performed.
Results	20d	Present results of sensitivity analyses.	Not applicable; no pooled synthesis was conducted.
Results	21	Present assessments of risk of bias due to missing results.	Not formally assessed because no pooled outcome was estimated. This limitation is acknowledged.
Results	22	Present assessments of certainty in the body of evidence.	No formal certainty rating was produced; the near-universal high PROBAST risk is reported and used to qualify conclusions.
Discussion	23a	Provide a general interpretation of results in the context of other evidence.	Discussion: Principal findings; validation; high reported performance; alternative algorithms; reproducibility; fairness and regulation.
Discussion	23b	Discuss limitations of the evidence included in the review.	Discussion: Interpretation of high reported performance; Limitations.
Discussion	23c	Discuss limitations of the review processes used.	Discussion: Limitations, including English-language restriction, search endpoint, restrictive Google Scholar query, and absence of formal agreement statistics.
Discussion	23d	Discuss implications for practice, policy, and future research.	Discussion: Geographic representation, fairness, and regulation; Implications for future research; Conclusion.
Other	24a	Provide registration information, including the register name and number.	Methods: Protocol and reporting; OSF registration DOI 10.17605/OSF.IO/7VN5X.
Other	24b	Indicate where the protocol can be accessed.	Methods: Protocol and reporting; linked OSF record.
Other	24c	Describe and explain amendments to the information provided at registration or in the protocol.	Methods: Protocol and reporting; revised title, operational scope, multi-omics definition, and omics-adjacent classification are disclosed.
Other	25	Describe sources of financial or non-financial support and the role of funders.	Declarations: Funding (no specific funding received).
Other	26	Declare competing interests of review authors.	Declarations: Competing interests (none declared).
Other	27	Report availability of template forms, extracted data, analytic code, and other materials.	Declarations: Availability of data and materials; Supplementary Data File 1; LaTeX source and R figure-reproduction files are included in the submission package.

Checklist wording adapted from PRISMA 2020 under the Creative Commons Attribution 4.0 licence. Reference: Page MJ, McKenzie JE, Bossuyt PM, et al. The PRISMA 2020 statement: an updated guideline for reporting systematic reviews. *BMJ*. 2021;372:n71. doi:10.1136/bmj.n71.
